# Excessive internet use as a risk factor for suicide ideation among university students in Malawi: A cross‐sectional study

**DOI:** 10.1002/hsr2.2194

**Published:** 2024-06-18

**Authors:** Thokozani Mzumara

**Affiliations:** ^1^ Department of Ophthalmology Mzuzu Urban Health Centre, Mzimba North District Hospital Mzuzu Malawi

**Keywords:** adolescent mental wellbeing, internet addiction, mental disorder, mental health, smartphone addiction, social media, suicidal thoughts

## Abstract

**Background:**

Covid 19 has fast‐paced the use of technological innovations, mainly the internet. However, Internet use can lead to several behavioral and psychological conditions, such as cyberbullying and distorted relationships, which could lead to suicide ideation. Suicide is the second leading cause of death among young adults.

**Aim:**

To assess the association between Internet addiction and suicide ideation among university students in Malawi. Furthermore, to assess the factors associated with suicidal thoughts among Malawian college students who surf the World Wide Web.

**Methods:**

This cross‐sectional study utilized secondary research and used data available from https://data.mendeley.com/drafts/xbfbcy5bhv. Internet addiction was measured using the Internet Addiction Test. The dependent variable includes suicide ideation. Binary logistic regression was used to analyze the relationship between the dependent and independent variables. The value of *p* < 0.05 was considered statistically significant.

**Results:**

Out of the 620 participants, 514 (82.2%) were aged between 15 and 24. The majority were males 401 (64.7%). The mean IAT score was 46.08 (SD = 14.60). The IAT score was 44.81 (SD = 13.85) among males and 48.40 (SD = 15.65) among females (*p* = 0.003). About 341 (55%) of students use the internet excessively. Suicide ideation was prevalent among 101 (16.3%) of the students. Suicide ideation was significantly associated with internet addiction. (*p* < 0.001). The odds of developing suicidal thoughts increased about 3 times among excessive internet users compared to average users (OR = 2.91, 95% CI = 1.213–7.018). However, age, gender, discipline, and year of study were not associated with suicide ideation.

**Conclusion:**

The study suggests that internet addiction affects suicide ideation mainly through distorting social relationships. School settings should increase awareness regarding the safe use of the internet to ensure a balance between online and real‐life interactions and curb suicide.

## 1 INTRODUCTION

The emergence of Web 2.0 has changed the landscape of the Internet, allowing for more web applications that encourage social interaction on the Internet.^1^ The Internet is a medium that can be used for different online activities, including gaming^2^, ^3^; social media usage^4^, ^5^, ^6^; and pornographic use.^7^ As a result, internet addiction can refer to a variety of online behaviors, and many measures of internet addiction (specific and generic) have been created.^8^, ^9^, ^10^, ^11^

Web 2.0 has spread the use of the internet with much hype, especially in higher education institutions in developing countries such as Malawi. Unfortunately, so many negative attributes can come along with this versatile technology. For instance, the proliferation of the internet and social media has exposed many youths to multiple negative effects. Makori, Agufana.^12^ Although technological advancements have provided many conveniences in terms of communication, especially among students, they have also exposed them to several emotional and safety threats. One of the most pressing issues regarding the innovation of the internet is the myriad of psychological problems grouped as depression.^13^ Arguably, the internet and social media have changed the culture of self‐expression where individuals who feel anonymous hide behind their digital screens and attack others in countless ways. The Internet has successfully dissolved the physical boundaries of communication and interaction.^14^

Internet addiction is a common phenomenon and refers to excessive use of the internet, which is characterized by uncontrollable urges associated with access to the internet.^15^ Internet addiction has been linked to higher rates of impulsivity, including several common mental disorders such as depression, anxiety, and loneliness, as well as suicide ideation and attempts.^15^ Ideally, loneliness can lead to internet surfing and searching for a company in pursuit of self‐worth.

Suicide is very common among youth (Meter et al., 2022). Currently, suicide among youth has emerged as a public health concern.^16^ Suicide behavior is an umbrella term that includes Suicide Ideation (SI), suicide plans, and suicide attempts.^16^ Previous research found that SI is experienced by one‐fifth of medical students in Africa. Some of the factors associated with SI in the African region include female gender use of alcohol and depression.^17^ Suicide ideation includes thoughts about ending one's life. Suicide thoughts are strongly linked to death by suicide. Globally, factors associated with suicide vary over space and time. Unfortunately, suicide cases are on the rise despite interventions designed to curb the social issue. The prevalence of suicide ideation was recorded at 7.2% in a nationally representative survey in Malawi and is higher than in 10 other lower‐income countries.^18^ However, there is no study reporting suicide ideation among youth in school settings.

A study conducted among Chinese adolescents found a strong link between suicidal ideation and problematic internet addiction.^19^ In contrast, a study in Korea found using the Internet was associated with lower levels of suicidal thoughts based on the mediation effect of the Internet to reduce depression through increased social relationship satisfaction among adults.^20^ There is a variation in the association between internet addiction and suicidal thoughts across contexts. It is widely accepted that adults use the internet differently from the youth who have championed this technology, especially in university settings.

Regardless of the poor information communication technology (ICT) infrastructure in Malawi, the adoption and use of the Internet are high among the student community (Chaputula et al., 2012). However, the literature suggests that excessive use of the Internet is strongly linked to chronic mental disorders. Among Malawian youth, sources of mental health issues emanate from four main pillars, including relationship issues, poverty‐related socioeconomic situations, and a lack of discussion on mental health issues (Jumbo et al., 2022). There is an obvious disparity related to gender and mental health. For instance, more males are at risk of developing mental problems than females, even though more females use the internet in Malawi (Manda et al., 2021). Nevertheless, it remains unclear how these negative effects of the internet induce thoughts of suicide and ultimately result in suicide among university students in Malawi.

University students experience a low quality of life, mainly emanating from academic pressure.^19^ Previous research has investigated the correlation between SI and IA among school‐going youth, and the majority of studies are conducted in Asia;^19^, ^21^, ^22^ Hang et al., 2024; Huang 202^23^. Despite the rapid proliferation of mobile technology among African youths,^24^ there is a dearth of research on suicide ideation and Internet addiction in African contexts. Therefore, this paper aims to examine the relationship between internet addiction and suicide ideation among university students in Malawi.

## 2 METHODS

### 2.1 Design

This cross‐sectional study was conducted as a secondary study and utilized data available online from https://data.mendeley.com/drafts/xbfbcy5bhv. the cross‐sectional survey employed a structured questionnaire that was distributed through Google Forms to various students across the country. The study included students from across 13 academic institutions, including the College of Medicine, the Malawi Polytechnic, the Malawi University of Science & Technology, the National College of Information Technology, the Malawi College of Accountancy, and the Catholic University of Malawi in the South; the Chancellor College and Domasi College of Education, in the East; the Malawi Institute of Management, Nalikule College of Education, Daeyang University, Natural Resources College, and Lilongwe University of Agriculture and Natural Resources in the Center; and Mzuzu University in the North (Manda et al., 2021). The data was collected from May to July 2018.

### 2.2 Inclusion and exclusion criteria

The study included data extracted from undergraduate students. However, as an exclusion criterion, the paper removed 364 cases with missing values on sociodemographic variables. Missing values on other variables, such as components of the internet addiction test, were replaced by the mode of the variable.

### 2.3 Dependent variable

Suicide ideation was the dependent variable. Suicide ideation was ascertained by answering the question “While using the internet, did you have thoughts of killing yourself? (Yes/No)”. It was extracted from the WHO self‐reporting questionnaire used to determine probable causes of mental health (Manda et al., 2021).

### 2.4 Independent variable

Internet addiction was answered by considering the 20 items stipulated under the internet addiction test (IAT), which assesses internet and social media use (Manda et al., 2021). The IAT included a six‐point Likert scale ranging from O does not apply to 5, meaning always. The total IAT test categorized internet users into various categories, including nonfrequent internet (less than 20 scores), average online user (20–39), excessive internet usage (40–69), and significant problem experience (70–100). Age was recoded into categories 15–24, 25–34, and 35 years and older. Gender was recoded as one for males and two for females. Discipline was split into classifications, namely the sciences, and the humanities and social sciences were coded as 1 and 2, respectively.

### 2.5 Analysis

The study data was input into the Statistical Package for Social Sciences, version 27. The descriptive statistics used in the article were mean and standard deviation, percentage, and proportions. To visually represent the data, the study employed pie charts and graphs. For univariate analysis, we employed chi‐square to determine the relationship between dependent and independent variables. We then used a binary logistic regression model to analyze the influence of the independent factors on the dependent variable. A value of *p* < 0.05 was considered statistically significant.

### 2.6 Ethics

Ethical approval by an ethical review board was waived due to the nature of the study since it used publicly available data from a previous survey. The survey was ethically approved and adhered to the Declaration of Helsinki.

## 3 RESULTS

The study recruited 620 undergraduate students from across the universities and colleges in the country. Out of the 620 participants, 514 (82.2%) were aged between 15 and 24. There were more males 401, 64.7%) than females. According to the discipline, the majority of students were engaged in studies related to science as a discipline, 77.4% (480). Based on the classification of Internet addiction, 38 (6%) were problematic Internet users, 341 (55%) were excessive Internet users, and 241 (39%) were average users.

The mean IAT score in the review was 46.08 (SD = 14.60). The IAT score was 44.81 (SD = 13.85) among males and 48.40 (SD = 15.65) among females. An independent *t* test showed that the difference in mean IAT score according to gender was statistically significant *t* (618) = −2.945, *p* = 0.003. According to categorization, the majority of participants 341, (55%) use the Internet excessively.

### 3.1 Prevalence of suicide ideation and associated factors

About, 101 pupils (16.3%) reported having suicide thoughts. Suicidal thoughts were reported by about 9 (23.7% of 38) students classified as problematic internet users, compared to 23 (9.5% of 241) ordinary internet users. Accordingly, the Chi‐square test found a statistically significant correlation between Suicide Ideation and Internet Addiction (*p* < 0.001). Furthermore, a higher number of girls, 40 (18% of 290), had suicidal thoughts than males, 61 (15% of 401). However, the gender difference was not statistically significant (*p* = 0.406). Furthermore, there was no statistically significant link between the year of study and suicidal thoughts (*p* = 0.348).

## 4 RISK FACTORS OF SUICIDE IDEATION

Logistic regression was used to investigate the impact of internet addiction, age, gender, discipline, and degree of education on the risk of suicidal thoughts. The model was statistically significant: *x* (7) = 18.371, *p* = 0.01. The model accounted for 5% (Nagelkerke R square) of the variation in suicide thoughts. The model successfully predicted 83.7% of the instances, with a sensitivity of 100% and a specificity of 0%. Excessive internet users were three times more likely to have suicidal thoughts than ordinary users (OR = 2.91, 95% CI = 1.213–7.018). Suicidal thoughts were unrelated to age, gender, discipline, or year of study (Table 1).

**Table 1:** Risk factors of suicide ideation.

			95% C.I. for EXP(B)	
Independent variables	*p* value	Exp(B)	Lower	Upper
Year of study	0.250			
Year of study (1)	0.718	1.108	0.636	1.929
Year of study (2)	0.279	.725	0.405	1.298
Discipline (1)	0.615	.878	0.528	1.459
Age group	0.993			
Age group (1)	0.907	1.097	0.232	5.187
Age group (2)	0.915	1.093	0.213	5.599
Sex (1)	0.432	1.205	0.757	1.917
Internet addition	0.001			
Internet addition (1)	0.017	2.918	1.213	7.018
Internet addition (2)	0.684	1.182	0.529	2.641
Constant	0.243	2.906		

## 5 DISCUSSION

In this study, students who used the internet excessively were more likely to experience suicidal thoughts compared to general users, similar to previous research.^19^, ^21^, ^22^, ^25^ Kuang 2020.^23^ The results of the study are unsurprising considering that the youth in sub‐Saharan Africa face many systemic challenges despite technological advancements.^26^ In this region, the youth get stressed about rapid changes in family structure and the socioeconomic landscape. The results of this review suggest that internet content could inspire thoughts of suicide, where students may start holding positive views about suicide due to exposure to harmful online information.^21^ The results of this study could also be explained by the proliferation of smartphones that have wifi functions and easy access to the Internet.^27^ Therefore, the study calls for appropriate censorship and other gatekeeping mechanisms to avoid SI and death by suicide.

In South Africa, the prevalence of SI was higher than in the current report.^28^ The prevalence of SI was 12.1% in a college in China.^21^ Moreover, the prevalence of SI was 37%. among students at vocational training schools in Hunan province in China.^23^ However, in this report, the prevalence of SI was higher than in previous studies done in Malawi. For instance, the prevalence of suicide ideation was reported at 7% in a nationally representative sample consisting of people aged 18–69 (penguin and Pelzer, 2021). Another study found that the prevalence of SI among hypertension and diabetes patients in Malawi was 2%.^29^ However, this is the first study to assess the prevalence of SI among the student population. The difference could be explained by the sample composition in terms of age and setting. The high prevalence of suicidal ideation in our study can be explained by the high levels of stress from academic environments, as evidenced by a large number of first‐year students who are not well adapted to the demands of university life. The results of this study are not alarming considering that the utilization of professional care following suicide ideation among Malawians is low, recorded at about 20%. Pengpid, Peltzer^18^ Furthermore, there is inadequate knowledge of tackling mental health issues in the African region.^30^ In addition, Malawi has limited mental health services to tackle the psychological issues facing the population (Udedi 2016). Our study's findings call for the provision of mental health services, including counseling, within school premises.

In the present study, there was no association between SI and sex similar to a study in China.^21^ In addition, a study conducted in South Africa reported that SI was associated with gender, similar to previous research.^31^ We attribute the difference to variations in socioeconomic status and ways of life across cultures.

The average IAT score in this study was 46.08 (SD = 14.60), similar to a recent study conducted among Egyptian students.^32^ Nevertheless, the average Internet addiction test was higher than previous reports elsewhere.^33^, ^34^ The high IA test score in the study is not surprising considering that academic stress is one of the causes of mental disorders.^32^ again, we attribute the difference to variations in instruments used to measure IA. For instance, this study used the IAT, while Shen and colleagues employed the revised Chinese Internet Addiction Scale. In part, the high prevalence of internet addiction due to the proliferation of mobile technology has taken Africa by storm, leading to uncontrollable use of the internet. The results of this paper call for awareness campaigns about the harmful effects of excessive internet use to avoid internet addiction among youth in institutions of higher learning. The addiction to the World Wide Web can lead to isolation from socializing with friends and loneliness, resulting in a mental breakdown. Hence, IA has a profound effect on neuroticism and psychoticism, leading to anxiety, depression, and eventually suicide ideas and attempts. Shinetsetseg et al.,.^22^ Indeed, Internet addicts are known for various personality problems, mainly emanating from time‐consuming mannerisms, resulting in a feeling of timelessness and destruction of people's lives.^32^

This study found that the IAT score was higher in females than males. In contrast, previous studies found a strong link between males and Internet Addiction^13^, ^33^ The findings of our study are expected considering that more females use the internet unlike males in Malawi (Manda et al., 2021). We attribute the difference to the use of the internet through mobile technology, which has eased access to social media applications such as Facebook, and Instagram, which appeal more to women and their intrinsic need to express themselves. Traditionally, the majority of women in Malawian culture are considered talkative. The data extracted in this study did not investigate the distribution of usage of the World Wide Web, but the mobile technocrats have intertwined social media platforms with the same ones that made the World Wide Web famous.

### 5.1 Limitations

The study is not without drawbacks. The study did not include an assessment of suicide attempts and suicide planning among the group. The study did not include other behavioral features such as drug use, alcohol use, smoking history age of initiation, or family structure. Furthermore, the study relied on self‐reported data and hence could be prone to bias. As a limitation, the study did not examine the association between SI and physical factors such as Body mass index, obesity, hypertension, and diabetes health risky behaviors such as smoking and drinking

### 5.2 Conclusion

The majority of Malawian youth experience suicidal ideation and use the internet excessively, suggesting that there is a high degree of loneliness and a lack of social and peer support among in‐school adolescents. The paper recommends a revamp of psychological and mental health services within university settings. Furthermore, university management should consider scaling up the formation of debate clubs and student communities to encourage a platform for real social support and discussion of mental health issues to curb suicide ideation and suicide attempts among students. Most significantly, school management and internet service providers can leverage the internet to ensure the gatekeeping of suicidal messages and information through the World Wide Web.

## AUTHOR CONTRIBUTIONS

**Thokozani Mzumara**: Conceptualization; investigation; writing–original draft; methodology; visualization; writing–review and editing; formal analysis; data curation.

## CONFLICT OF INTEREST STATEMENT

The author declares no conflict of interest.

## ETHICS STATEMENT

Ethical review was waived due to the nature of the study.

## TRANSPARENCY STATEMENT

The lead author Thokozani Mzumara affirms that this manuscript is an honest, accurate, and transparent account of the study being reported; that no important aspects of the study have been omitted; and that any discrepancies from the study as planned (and, if relevant, registered) have been explained.

## Data Availability

The data is available through https://data.mendeley.com/drafts/xbfbcy5bhv.
